# Cross-cultural adaptation and psychometric evaluation of the Chinese version of the short version of the Cognitive Reserve Index Questionnaire (s-CRIq): a cross-sectional study

**DOI:** 10.1186/s12888-026-08041-w

**Published:** 2026-04-04

**Authors:** Zhen-zhen Chu, Xue-xue Chen, Ming-shan Qi, Jie-qiong Ren, Xiao-juan Wang

**Affiliations:** 1 School of Nursing, Ningxia Medical University, Yinchuan, China; 2 Neurology Department, People’s Hospital of Ningxia Hui Autonomous Region, Ningxia Medical University, Yinchuan, China

**Keywords:** Cognitive reserve, Cultural adaptation, Reliability, Validity, China

## Abstract

**Background:**

Assessing cognitive reserve (CR) is crucial for identifying the risk of cognitive impairment, predicting disease progression, and informing targeted interventions. However, the Cognitive Reserve Index Questionnaire (CRIq), a widely used tool for evaluating CR, has practical limitations including its length, complex collection requiring interviewer involvement, and time burdens for participants and interviewers. To address these, a self-administered short-form version (s-CRIq) was updated in 2023, which retains the original scale’s core structure and dimensions while significantly improving convenience and efficiency. This study aimed to develop a Chinese version of the s-CRIq (s-CRIq-C) through rigorous cross-cultural adaptation and evaluate its psychometric properties.

**Methods:**

Followed Beaton’s guidelines for cross-cultural adaptation to translate the s-CRIq-C. Seven experts evaluated the content validity index and translation validity index. Independent samples *t*-tests were used to assess gender differences, while one-way ANOVA with post-hoc analyses was employed to examine age group differences. To verify the construct validity of the s-CRIq-C, Pearson correlation analysis was performed to assess the relationship between the total scale score and the three dimension scores. Concurrent validity was tested using the full-length CRIq as the criterion to further confirm its construct validity. Reliability was evaluated through internal consistency and test-retest reliability.

**Results:**

A total of 315 healthy adult participants were included. The mean total CRI score was 94.28 ± 14.94, indicating a medium level of CRI. The total CRI score showed strong correlations with the three dimensions—CRI-Education (*r* = 0.818), CRI-WorkingActivity (*r* = 0.812), and CRI-LeisureTime (*r* = 0.819)—while moderate to weak correlations were observed among the dimensions themselves. Concurrent validity analysis revealed a high correlation between the s-CRIq-C and the long version of CRIq (*r* = 0.932, *p* < 0.001). The s-CRIq-C exhibited good reliability, with a Cronbach’s α coefficient of 0.777, McDonald’s ω coefficient of 0.780, and test-retest reliability of 0.968.

**Conclusions:**

The s-CRIq-C demonstrated good reliability and validity, supporting its utility as a brief and dependable tool for assessing CR in both clinical and scientific investigations.

**Trial registration:**

The trial was registered at the Chinese Clinical Trial Registry with the registration number ChiCTR2500104640. Registered on 20 June 2025.

## Background

Cognitive impairment has emerged as a significant global public health concern, with approximately 55 million individuals affected by dementia worldwide. This number is increasing at an alarming rate—projected to reach 78 million by 2030 and 139 million by 2050 [1]. This condition not only results in functional disability [2] and diminished quality of life [3] but also imposes substantial societal and economic burdens, contributing to a global annual economic loss estimated at 1.3 trillion US dollars [4]. China, with its rapidly expanding elderly population, has over a quarter of individuals experiencing cognitive dysfunction [5].

Cognitive reserve (CR) refers to the brain’s adaptive capacity to optimize neural network functioning (efficiency, capacity, and flexibility), explaining interindividual variability in susceptibility to age- or pathology-related cognitive deficits [6]. The CR theory posits that a higher level of CR mitigates cognitive decline through compensatory mechanisms and neural network reorganization, distinct from the static and passive nature of brain reserve (referring to the brain’s structural capacity or neurobiological resources like neurons and synapses) [7]. The neural mechanisms underlying cognitive reserve are primarily manifested through the dual modes of neural reserve and neural compensation [8]. Both modes play crucial roles in mitigating cognitive decline through distinct pathways. Neural reserve refers to innate individual differences in the efficiency, capacity, or flexibility of neural networks within healthy brains, allowing individuals with higher levels of CR to maintain superior cognitive performance despite equivalent neuropathological burden [9]. Neural compensation refers to the brain’s ability to recruit alternative neural networks or reorganise functional connectivity in response to deficits caused by injury or disease affecting primary task-related networks. Individuals with higher levels of CR exhibit enhanced capacity to utilise compensatory neural pathways, thereby preserving cognitive function in response to cerebral alterations [10]. The dynamic capacity of CR explains why individuals with comparable levels of neuropathological damage may exhibit significantly different cognitive outcomes [11]. Specifically, those with a higher CR demonstrate a reduced risk of cognitive impairment [12, 13], slower cognitive decline [14, 15], and enhanced post-brain injury recovery [16]. Consequently, early CR assessment holds critical clinical significance for identifying high-risk populations, predicting disease progression, and guiding personalized interventions [17].

CR is a lifelong accumulation process [18] influenced by various factors such as education level, socioeconomic status, physical activity, social interactions, occupational characteristics, innate intelligence, and multilingual abilities [19–21]. Notably, gender factors uniquely influence the development of CR. According to social role theory, gender-differentiated educational experiences, career choices, and social expectations contribute to the distinct cognitive stimuli that individuals receive, ultimately resulting in different opportunities and pathways for men and women to accumulate CR over their lifetimes [22]. As CR is a theoretical construct, no standardized direct measurement method currently exists. Common evaluation approaches include socio-behavioral proxies (using either individual or combined), residual approaches [23, 24], and functional imaging techniques [25, 26]. Currently, research tends to favor the use of combined socio-behavioral proxy indices due to two main advantages: first, they are more practical compared to complex residual quantification methods or expensive functional imaging technologies; second, the multidimensional proxy index can more comprehensively reflect the core concept of the original theoretical CR model, thus demonstrating significantly improved evaluation validity compared to single-proxy measures [27].

Various CR assessment tools have been developed for diverse populations and proxy indicator combinations. Among them, the Cognitive Reserve Index Questionnaire (CRIq) [28], Cognitive Reserve Questionnaire (CRQ) [29], Cognitive Reserve Scale (CRS) [30], and Pertinent Activities Practice in Adults (PAPA) [31] are applicable across young, middle-aged, and older adults. Meanwhile, the Lifetime of Experiences Questionnaire (LEQ) [32], Premorbid Cognitive Abilities Scale (PCAS) [33], and Retrospective Indigenous Childhood Enrichment Scale (RICE) [34] are tailored specifically for older adults. Additionally, the Cognitive Reserve Assessment Scale in Health (CRASH) [35] is designed for individuals with severe mental illness, whereas the Cognitive Reserve Potential Questionnaire (CRPQ) [36] assesses cognitive reserve in adolescents. Recent reviews indicate that the CRIq is the leading tool for assessing CR in both healthy and clinical populations [37]. Initially introduced by Nucci et al. in 2012, the CRIq assesses lifespan CR accumulation and has been translated into more than a dozen languages. In 2023, Mondini et al., from the same research group, updated and enhanced the online short version of the Cognitive Reserve Index Questionnaire (s-CRIq) [38]. Both versions encompass three dimensions: education, working activities, and leisure time. CRI-Education includes educational attainment and structured courses lasting more than six months. CRI-WorkingActivity covers occupational history during adulthood and total years of employment. CRI-LeisureTime comprises six items related to activities pursued during leisure time and their duration, such as reading newspapers or magazines, attending performances or exhibitions, traveling, and engaging in physical exercise. The s-CRIq was developed to optimize the original CRIq for enhanced applicability in modern research and clinical practices. Multifaceted innovation through streamlining and online improvements: (1) CRI-Education: simplified operational processes by directly selecting the highest education level (the CRIq requires entering total years of education). (2) CRI-WorkingActivity: improved precision through the International Standard Classification of Occupations (ISCO-08) implementation for selecting specific occupations from approximately 6,000 options (the CRIq selects working activity levels). (3) CRI-LeisureTime: the scale was refined from 17 to 5 items using item response theory and confirmatory factor analysis, with one additional item included, resulting in a total of 6 items. (4) Algorithm adjustments: the s-CRIq retains the long version’s calculation logic and CR classification standards but modifies the CRI-WorkingActivity scoring method to prevent potential underestimation in cases of multiple work experiences. (5) The s-CRIq employs a self-administration model (the CRIq suggests semi-structured interview). Although direct tests of the s-CRIq’s reliability and validity are lacking, its validity is supported by two aspects: alignment with the CRIq’s theoretical framework and the overlap area between empirical density functions, as quantified by the Overlapping Index. Overall, the s-CRIq enhances practical utility and operational efficiency while preserving core assessment functions, which not only improves the accuracy and timeliness of data collection, but also makes the tool easier to apply in various scenarios, including rapid screening and large-scale epidemiological investigations. However, there is currently no Chinese version of the s-CRIq available for use in Chinese-speaking populations. Therefore, this study aims to develop the Chinese version of the s-CRIq (s-CRIq-C) through translation and cross-cultural adaptation, and assess the reliability and validity of the s-CRIq-C.

## Methods

### Design

A cross-sectional survey was conducted to evaluate the psychometric properties of the Chinese version of the s-CRIq (s-CRIq-C). The study comprised two sequential phases: (1) systematic translation and cross-cultural adaptation of the s-CRIq into Chinese, followed by (2) comprehensive validation in healthy adults.

### Participants

From June to July 2025, using a convenience sampling method to investigate healthy adults with a wide range of demographic characteristics. The inclusion criteria were as follows: (1) native Chinese speakers with normal communication skills, (2) healthy adults over 18 years of age, and (3) signed informed consent. The exclusion criteria were as follows: (1) presence of neurological disorders (e.g., Alzheimer’s disease, Parkinson’s disease, stroke) that may affect the cognitive test; (2) history of severe head trauma, learning disabilities, intellectual disability, or major psychiatric disorders (e.g., schizophrenia, bipolar disorder), or current use of antipsychotic medication.

The sample size was determined based on psychometric theory principles proposed by Nunnally and Bernstein [39], which recommend a participant-to-item ratio ranging from 5:1 to 10:1 for scale validation studies. Since the s-CRIq comprises 9 items, an initial sample size of 90 participants was determined. Accounting for a 20% invalid response rate, the final estimated sample size was at least 113 participants.

### Instruments

#### The Cognitive Reserve Index questionnaire (CRIq)

The CRIq [28] is a comprehensive instrument designed to evaluate an individual’s CR. It retrospectively collects cognitive stimulus activity accumulated over a lifetime. Developed by Nucci et al. in 2012, the scale collects demographic information (e.g., name, date and place of birth, age, place of residence, nationality, and civil status) and comprises 20 items organized into three dimensions: CRI-Education (2 items), CRI-Working Activity (1 item), and CRI-Leisure Time (17 items). The total Cognitive Reserve Index (CRI) and subdomain scores were computed for each participant using the standardised calculation tool developed by Nucci et al. (https://www.cognitivereserveindex.org/paperadm.html). This tool automatically applies an age-based standardisation procedure, converting raw CRIq scores into age-adjusted values with a mean of 100 ± 15. This standardisation procedure was applied to eliminate age-related effects and facilitate cross-age-group comparisons. The CRI score is classified into five levels: low (< 70), medium-low (70 ~ 84), medium (85 ~ 114), medium-high (115 ~ 130), and high (> 130), with higher scores denoting greater CR capacity. The CRIq demonstrated a Cronbach’s α coefficient of 0.73, indicating good internal consistency. The present study adopted the Chinese version of the CRIq developed by Cao et al. [40] in 2020, which demonstrated a Cronbach’s α coefficient of 0.68 in adults aged 18 years and older.

#### The Chinese version of the online short version of the Cognitive Reserve Index questionnaire (s-CRIq-C)

The s-CRIq-C collects demographic information (e.g., name, sex, date and place of birth, place of residence, nationality) and comprises 9 items in three dimensions: CRI-Education (2 items), CRI-WorkingActivity (1 item), and CRI-LeisureTime (6 items). The total CRI score and dimension scores for all participants were computed automatically via a standardized Excel spreadsheet (with embedded formulas) provided by Professor Mondini, into which raw responses were entered. The levels of CRI score classifications remain consistent with the CRIq.

### Translation and cross-cultural adaptation

In this study, written authorization was obtained from the original author (Sara Mondini) via email. The translation process followed Beaton’s guidelines for cross-cultural adaptation [41]. All researchers involved in the translation had no prior exposure to the scale and had passed China’s College English Test Band 6 (CET − 6), demonstrating good bilingual competency. The detailed procedures were as follows:

- **Step 1: Forward translation**: The s-CRIq was independently translated into two Chinese versions (T1 and T2) by two native Chinese-speaking nursing graduate students. The first translator (T1) was a nursing graduate student with clinical experience and familiarity with the scale’s content. The second translator (T2) was a nursing teacher pursuing graduate studies but lacked clinical experience and had limited understanding of the scale’s purpose and conceptual meaning.
- **Step 2: Synthesis of the Chinese translation**: A doctor of Nursing consolidated T1 and T2, subsequently refining them through panel discussions on disparities in terminology, sentence structures, semantic equivalence, and cultural nuances. This process led to the development of the Chinese version of T-12.
- **Step 3: Back translation**: A Ph.D. in English who teaches at a medical school back-translated Chinese T-12 into English.
- **Step 4: Expert committee review**: The Delphi method was employed to evaluate the linguistic consistency, cultural relevance, and content validity of T-12, using a 4-point Likert scale. Seven experts, comprising five individuals with doctoral degrees, two with master’s degrees, including one neurologist, and six nursing specialists specializing in geriatrics or cognitive fields, were invited to participate in two rounds of evaluations conducted via email. Subsequently, the research team incorporated expert feedback to revise the questionnaire, producing the draft s-CRIq-C.
- **Step 5: Pre-testing**: In June 2025, a convenience sample of 30 participants (excluded from the main study cohort) completed the pre-testing. Following informed consent procedures, the research objectives and significance were explained to the participants. Subsequently, researchers conducted face-to-face interviews to assess comprehension and relevance. Item descriptions were further refined based on participant feedback.
- **Step 6: Submission and appraisal of all written reports by developers/committee**: All documentation—including T-12, its back-translation, expert reviews, and pre-testing revisions—was compiled into a report and submitted to the original scale developers for validation.

### Data collection

To accommodate the needs of localized research in China, this study utilized Questionnaire Star, a commonly used domestic online survey platform, for data collection using a convenience sampling method. Participants were able to access the questionnaire directly on their smartphones without the need for a specific URL or account login. A comprehensive explanation of the study, including its objectives, importance, and guidelines, was presented on the initial page. Electronic informed consent was obtained via a binary interface (“Agree to participate”/“Decline to participate”). Platform settings restricted access to the questionnaire, permitting progression only upon selecting “Agree to participate”. Eligibility criteria were enforced through three screening questions assessing: ① history of severe head trauma, learning or intellectual disabilities; ② current or past neurological/psychiatric conditions; and ③ antipsychotic medication use. Participants who answered “No” to all items were deemed eligible and allowed to proceed to subsequent questionnaire sections.

### Quality control

Rigorous quality control protocols were implemented in this research to ensure the reliability of the data. Prior to data collection, electronic questionnaires were utilized jump logic and forced completion. Subsequently, during data entry, invalid questionnaires were excluded based on the following criteria: ① questionnaires with s-CRIq-C completion times < 3 min (below the pre-testing average of 3–5 min); ② questionnaires with highly consistent or contradictory responses.

### Data analysis

Statistical analyses were performed using Excel, IBM SPSS 27.0 and Jamovi 2.6.44. Continuous variables were presented as mean ± standard deviation for normally distributed data or median (interquartile range, IQR) for non-normally distributed data, and categorical variables were presented as frequencies and percentages. For comparisons between groups, independent-sample *t*-tests were used for gender differences and one-way ANOVA for differences across age groups, provided that the data followed a normal distribution and met the assumption of homogeneity of variance. If the ANOVA results were significant, post-hoc tests were conducted. When these assumptions were violated, the Mann-Whitney *U* test for gender, and the Kruskal-Wallis test for age groups. A significant Kruskal-Wallis test was followed by post-hoc pairwise comparisons using Dunn’s test with Bonferroni correction. A two-sided *p* < 0.05 was considered statistically significant.

### Item analysis

Item analysis was conducted using item-total correlations [42]. Pearson correlation coefficients were employed to examine the relationship between each item of the scale and the total CRI score, retaining only items with correlation coefficients > 0.3 [43].

### Validity analysis

#### Content validity

Content validity was assessed by seven experts using a 4-point Likert scale (1 = not relevant, 2 = somewhat relevant, 3 = quite relevant, 4 = highly relevant). Two indices were calculated: the item-level content validity index (I-CVI), indicating the proportion of experts rating an item as relevant (3 or 4), and the scale-level content validity index (S-CVI), comprising the universal agreement index (S-CVI/UA) - the percentage of items rated 3 or 4 by all experts - and the average agreement index (S-CVI/Ave) - the mean of all I-CVI scores. Following established standards, acceptable validity thresholds were defined as I-CVI ≥ 0.78, S-CVI/UA ≥ 0.80, and S-CVI/Ave ≥ 0.90, ensuring robust content validity [44].

#### Translation validity

The translation’s cultural applicability and linguistic accuracy were evaluated through expert consultation. Each item was evaluated using the translation validity index (TVI) and received suggested modifications. The TVI, a tool developed by Tang and Dixon [45] based on the CVI, employs a 4-point Likert scale: 1 point for “inconsistent”, 2 points for “relatively inconsistent, requiring substantial modifications”, 3 points for “consistent, requiring minor modifications”, and 4 points for “completely consistent”. The scale-level TVI (S-TVI) was determined by calculating the percentage of items rated 3 or 4 out of the total number of items. Ultimately, the S-TVI achieved 100%, indicating unanimous expert acknowledgment of the translation quality.

#### Construct validity

In this study, Pearson correlation analysis and concurrent validity were employed to assess the construct validity of the scale, rather than exploratory factor analysis (EFA) or confirmatory factor analysis (CFA), for the following reasons: firstly, the dimension CRI-WorkingActivity consists of a single item, which does not meet the minimum requirement of three items per dimension for EFA and CFA to ensure model identification conditions. Consequently, inter-item correlations or factor loadings cannot be computed for a single item, leading to unreliable results in the factor analysis model [46, 47]. Secondly, the original CRIq has undergone validation in various studies, exhibiting strong psychometric properties, and has been translated and adapted across various countries, demonstrating its robust construct validity. The s-CRIq utilized in this study comprises selected items from the original CRIq, with its content validity supported by expert evaluation and structural confirmation of the original scale. Hence, there is no necessity to re-conduct factor analysis.

#### Concurrent validity

Concurrent validity [48, 49] refers to the degree of association between a newly developed measurement tool and an established, widely accepted measurement tool (a gold standard or commonly used measure) assessed at the same time. Its purpose is to examine whether the new tool can serve as a substitute for or supplement to the existing measure. In the study, the full-length CRIq was employed to evaluate the concurrent validity of the s-CRIq-C. Strong correlation is denoted by a correlation coefficient ≥ 0.7, moderate between 0.5 and 0.7, low between 0.3 and 0.5, and negligible when less than 0.3.

### Reliability analysis

This study assessed reliability using measures of internal consistency and test-retest reliability. Both Cronbach’s α coefficient and McDonald’s ω coefficient were calculated to provide robust estimates of internal consistency, with values > 0.70 indicating acceptable reliability [50, 51]. To assess test-retest reliability, 30 participants were randomly selected from the initial cohort to complete the s-CRIq-C again after two weeks. As recommended by Nunnally & Bernstein [39], a 2-week interval for test-retest is sufficiently long to avoid recall of previous responses by participants, yet sufficiently short to minimize the potential influence of health status changes on the outcomes. To ensure anonymity and confidentiality, each participant was assigned a unique, randomly generated code, thereby enabling the correlation of baseline data with follow-up assessments. Contact details were collected from the first 30 participants who consented to retake the questionnaire after two weeks, following which a new survey link was sent to them. The sample size was determined following the guidelines by Bujang et al. [52], considering a significance level (*α*) of < 0.05, statistical power (*β*) of ≥ 90%, and an expected intraclass correlation coefficient (ICC) of ≥ 0.5. A minimum of 30 participants was considered necessary to fulfil these criteria. The interpretation of ICC values followed established guidelines [53]: < 0.5 (poor), 0.5–0.75 (moderate), 0.75–0.9 (good), and > 0.90 (excellent).

## Results

### The translation and cultural adaptation

Following forward-backward translation, the s-CRIq-C was modified through panel discussions incorporating feedback from seven domain experts and 30 participants in pre-testing. All nine items were retained after adaption. Item 1 (Education Level): Classification was adjusted to align with China’s educational system, using standardized academic degree tiers for cultural relevance. Item 3 (Paid Work): Added operational definition: “All contractual work involving time investment for financial compensation (including part-time employment)” to clarify scope. Item 4 (Reading Newspapers or Magazines): Specified inclusion of “both print and digital formats” to reflect modern reading habits. Items 5–6 (Leisure Activities & Conferences, concerts or exhibitions): The examples were supplemented with activities that demonstrate higher cultural relevance to daily life in China. Item 8 (Reading Books): Specify “excluding textbooks or course-assigned readings” to maintain distinction from the CRI-Education dimension. Items 4–8: “All my life” was revised to “All the time” to better conform to Chinese linguistic conventions.

### Participant characteristics

A total of 321 questionnaires were enrolled, from which 315 valid responses were obtained, resulting in an effective response rate of 98.13%. The participants’ ages ranged from 19 to 93 years (mean age: 49.97 ± 21.45). Based on the full-length CRIq, all participants were categorized into three age groups: Young (19–44 years; *n* = 126, 27.03 ± 7.01), Middle-Aged (45–69 years; *n* = 97, 55.09 ± 6.51), and Elderly (70–93 years; *n* = 92, 75.97 ± 5.93). Detailed demographic characteristics can be found in Table 1.

**Table 1:** Demographic characteristics of participants

	Total **(*n*** **= 315)**	Young **(*n*** **= 126)**	Middle-Aged **(*n*** **= 97)**	Elderly **(*n*** **= 92)**		Male **(*n*** **= 142)**		Female **(*n*** **= 173)**
Gander, *n*(%) (male/female)	142/173 (45.1%/54.9%)	32/94 (25.4%/74.6%)	55/42 (56.7%/43.3%)	55/37 (59.8%/40.2%)		NA		NA
Age (years, M ± SD)	49.97 ± 21.45	27.03 ± 7.01	55.09 ± 6.51	75.97 ± 5.93		57.07 ± 19.15		44.13 ± 21.52
**Ethnic groups**, ***n*(%)**								
Han	214 (67.9%)	108 (85.7%)	55 (56.7%)	51 (55.4%)		96 (67.6%)		118 (68.2%)
Hui	66 (20.9%)	8 (6.3%)	32 (33.0%)	26 (28.3%)		35 (24.6%)		31 (17.9%)
Mongol	31 (9.8%)	6 (4.8%)	10 (10.3%)	15 (16.3%)		11 (7.7%)		20 (11.6%)
Others	4 (1.3%)	4 (3.2%)	0 (0.0%)	0 (0.0%)		0 (0.0%)		4 (2.3%)
**Educational level**, ***n*(%)**								
No formal schooling	18 (5.7%)	1 (0.8%)	2 (2.1%)	15 (16.3%)		8 (5.6%)		10 (5.8%)
Primary school	59 (18.7%)	7 (5.6%)	14 (14.4%)	38 (41.3%)		34 (23.9%)		25 (14.5%)
Junior high school	55 (17.5%)	8 (6.3%)	17 (17.5%)	30 (32.6%)		30 (21.1%)		25 (14.5%)
Senior high school	41 (13.0%)	5 (4.0%)	31 (32.0%)	5 (5.4%)		26 (18.3%)		15 (8.7%)
Associate degree	23 (7.3%)	6 (4.8%)	17 (17.5%)	0 (0.0%)		8 (5.6%)		15 (8.7%)
Bachelor’s degree	75 (23.8%)	59 (46.8%)	12 (12.4%)	4 (4.3%)		29 (20.4%)		46 (26.6%)
Master’s/ Doctoral degree	44 (14.0%)	40 (31.7%)	4 (4.1%)	0 (0.0%)		7 (4.9%)		37 (21.4%)

Table 2 displays the distribution of CRI levels by sex and age group. The data reveals that the majority of participants (63.8%, *n* = 201) displayed Medium CRI levels, with 23.5% (*n* = 74) showing Medium-Low levels.

**Table 2:** Classification of CRI levels categories according to gender and age groups

CRI levels	Total (*n* = 315)	Gender		Age			
		Male (*n* = 142)	Female (*n* = 173)	Young (*n* = 126)		Middle-Aged (*n* = 97)	Elderly (*n* = 92)
Low	14 (4.4%)	5 (3.5%)	9 (5.2%)	0 (0.0%)		0 (0.0%)	14 (15.2%)
Medium-Low	74 (23.5%)	43 (30.3%)	31 (17.9%)	8 (6.3%)		15 (15.5%)	51 (55.4%)
Medium	201 (63.8%)	75 (52.8%)	126 (72.8%)	116 (92.1%)		60 (61.8%)	25 (27.2%)
Medium-High	20 (6.3%)	14 (9.9%)	6 (3.5%)	2 (1.6%)		16 (16.5%)	2 (2.2%)
High	6 (1.9%)	5 (3.5%)	1 (0.6%)	0 (0.0%)		6 (6.2%)	0 (0.0%)

Table 3 displays the total CRI score and three dimension scores categorized by gender and age group. The mean total CRI score was 94.28 ± 14.94, indicating a medium level of CRI. Independent samples *t*-tests revealed significant sex differences in both CRI-WorkingActivity (*t* = 2.704, Cohen’s *d* = 0.318, *p* < 0.01) and CRI-LeisureTime subscores (*t* = -2.715, Cohen’s *d* = -0.313, *p* < 0.01). One-way ANOVA demonstrated significant age-related effects on total CRI score and three dimension scores. Specifically, the middle-aged group exhibited significantly higher total CRI score (103.46 ± 15.03) in comparison to the young (97.97 ± 6.85) and elderly groups (79.54 ± 11.79) (*F*_(2, 312)_ = 115.981, *p* < 0.001, *η²* = 0.426). Subsequent *post-hoc* analyses confirmed significant pairwise differences among all three age groups (all *p* < 0.001). Regarding dimension scores, the middle-aged group outperformed other age groups in CRI-Education (*F*_(2, 312)_ = 20.813, *p* < 0.001, *η²* = 0.118), CRI-WorkingActivity (*F*_(2, 312)_ = 78.127, *p* < 0.001, *η²* = 0.334), and CRI-LeisureTime (*F*_(2, 312)_ = 328.476, *p* < 0.001, *η²* = 0.678). Further *post-hoc* analyses revealed: (1) significant differences in CRI-Education between middle-aged vs. elderly and young vs. elderly groups (both *p* < 0.01); (2) significant pairwise differences in both CRI-WorkingActivity and CRI-LeisureTime among all age groups (all *p* < 0.01).

**Table 3:** Total CRI score and dimension scores categories by gender and age group

	Total (*n* = 315)	Gender				Age				
		Male (*n* = 142)	Female (*n* = 173)	*t*	*p*	Young (*n* = 126)	Middle-Aged (*n* = 97)	Elderly (*n* = 92)	*F*	*p*
CRI	94.28 ± 14.94	94.04 ± 16.94	94.48 ± 13.11	-0.256	0.798	97.97 ± 6.85	103.46 ± 15.03	79.54 ± 11.79	115.981	< 0.001
CRI-Education	94.39 ± 12.68	93.65 ± 12.45	94.99 ± 12.86	-0.929	0.354	97.02 ± 10.70	97.37 ± 14.51	87.63 ± 10.48	20.813	< 0.001
CRI-WorkingActivity	100.60 ± 14.44	103.10 ± 17.15	98.55 ± 11.41	2.704	0.007	97.33 ± 5.42	112.70 ± 15.04	92.33 ± 14.16	78.127	< 0.001
CRI-LeisureTime	91.68 ± 14.55	89.20 ± 15.77	93.71 ± 13.17	-2.715	0.007	100.90 ± 4.75	97.23 ± 11.10	73.21 ± 8.62	328.476	< 0.001

### Item analysis

In this study, item-total correlations analysis demonstrated that item 9 (“collecting the number of children participants had”) exhibited a negative correlation with the total score (*r* = -0.462), whereas all other items demonstrated positive correlations (*r* = 0.41–0.81, all *p* < 0.05). Among these, the weakest positive correlation (*r* = 0.41) was observed for the item “Do you engage in leisure activities at least three times a week during your free time?”, while the strongest correlation (*r* = 0.81) was found for “Please list the paid jobs (referring to the jobs where you devote your time and receive corresponding compensation according to the contract) you have held since adulthood (starting from the age of 18) and the duration of these jobs”. Further analysis of Item 9 indicated that participants with ≥ 3 children had a mean age of 68.35 ± 12.92 years, while those with ≤ 2 children had a mean age of 44.02 ± 20.26 years. Finally, no items were deleted during the item analysis phase, and all 9 items were retained.

### Validity analysis

#### Content validity and translation validity

The content validity of the s-CRIq was rigorously evaluated through a two-round Delphi expert consultation process. Seven domain experts independently rated each item using a standardized 4-point Likert scale. The I-CVI scores ranged from 0.86 to 1.00, while the S-CVI/UA was 0.93 and the S-CVI/Ave reached 0.99. The coefficient of variation (CV) for evaluation scores ranged from 0 to 0.23 (all values < 0.25). The S-TVI of the s-CRIq reached 100%.

#### Construct validity and concurrent validity

Pearson correlation analysis was performed to assess the associations between the total CRI score and its dimension scores. The results showed strong positive correlations between the total score and all three dimensions: CRI-Education (*r* = 0.818, *p* < 0.001), CRI-WorkingActivity (*r* = 0.812, *p* < 0.001), and CRI-LeisureTime (*r* = 0.819, *p* < 0.001). In contrast, moderate to weak correlations were found among the dimensions: CRI-Education and CRI-WorkingActivity (*r* = 0.537), CRI-Education and CRI-LeisureTime (*r* = 0.533), and CRI-WorkingActivity and CRI-LeisureTime (*r* = 0.457), all *p* < 0.001. Concurrent validity was assessed using the CRIq as the criterion measure. By correlation analysis, the s-CRIq-C was highly correlated with the target measure, with a strong correlation coefficient of *r* = 0.932 (*p* < 0.001).

### Reliability analysis

The Cronbach’s α coefficient was 0.777 and McDonald’s ω coefficient was 0.780, indicating that the s-CRIq-C demonstrated acceptable internal consistency. Furthermore, all 30 participants completed the second round of questionnaires, and the two-week test-retest reliability analysis demonstrated excellent temporal stability (*r* = 0.968).

## Discussion

The increasing prevalence of cognitive impairment has led to a growing recognition of its substantial impact on both individual quality of life and socioeconomic burdens [54]. Researchers are increasingly focusing on assessing CR before the onset of cognitive impairment to enable early intervention. While the CRIq is widely used, its considerable length may lead to respondent fatigue and affect compliance rates, particularly in large-scale epidemiological studies. This investigation introduces the s-CRIq as a tool to assess CR in individuals aged 18 and above. This instrument aims to provide a concise measurement tool for future clinical research, facilitating the efficient large-scale collection of CR data in our country.

The findings of this study demonstrate that the s-CRIq-C is a practical and efficient instrument for assessing CR in Chinese populations. Notably, the total CRI score exhibited strong correlations with its subscores, confirming that each proxy measure meaningfully contributes to the composite CR evaluation. In line with previous studies by Çebi et al. [55] and Cao et al. [40], inter-subscore correlations were low to moderate, underscoring the distinctiveness of each proxy domain.

The distribution of CRI levels in this study revealed that the majority of participants (63.8%) were categorized as moderate, with low and high levels being less prevalent, particularly among younger individuals. This trend aligns with findings from previous studies utilizing the original CRIq and research conducted in multiple countries [28, 40, 55, 56]. It is plausible that the younger individuals, who benefit from broader educational and occupational opportunities compared to older generations constrained by historical factors, tend to exhibit higher CRI levels [57]. In contrast, older individuals are more inclined towards lower CRI classifications. Furthermore, younger participants may not yet have achieved peak career complexity or engaged in a sufficient number of lifelong leisure activities, leading to predominantly moderate rather than high CRI levels in this group [58].

Consistent with previous studies [40], no significant disparity was found in total s-CRIq score and CRI-Education between males and females. However, men scored significantly higher than women in the CRI-WorkingActivity. The data from this study show that the proportion of men who have completed high school or higher education (49.30%) is lower than that of women (65.32%) (Table 1). This educational gap might predispose men to engage in longer and more continuous workforce participation, thereby contributing to their higher CRI-WorkingActivity scores. In contrast, women exhibited significantly higher scores than men in the CRI-LeisureTime. This discrepancy may be attributed to traditional Chinese gender norms, which shape occupational and leisure activity patterns, particularly among middle-aged and older adults. For instance, women tend to participate more in group-based leisure activities such as square dancing and community crafts [59, 60]. Moreover, the earlier statutory retirement age for women in China (typically 50–55 years), compared to men (60 years), may limit their accumulation of occupational experiences while expanding their leisure time [61]. Significant variations were observed in the total CRI score and the three dimension scores across different age brackets (*p* < 0.05). The older group exhibited significantly lower scores than the other age groups in all scores, which aligns with the original CRIq and results from multiple countries [28, 40, 55, 56]. This trend might be due to older individuals having fewer educational opportunities and being less involved in complex occupations. Additionally, age-related declines in physical capabilities and the burden of chronic ailments may lead to decreased engagement in leisure activities [62].

The item analysis indicated that item 9 (collecting the number of children participants had) demonstrated a negative correlation with the total score (*r* = -0.462), while all other items exhibited positive correlations. This discrepancy may be attributable to the observation that participants with ≥ 3 children in this study were predominantly older (mean age 68.35 ± 12.92, *n* = 77) and, owing to historical constraints (e.g., the absence of fertility restrictions in China prior to 1980), likely faced limited career opportunities and inadequate childcare support (e.g., lack of access to postnatal carers or domestic helpers). Consequently, substantial time devoted to child-rearing may have adversely impacted their scores across other dimensions of the scale. Notably, the CRIq—which includes an identically phrased item (positively scored)—has been translated and retained in over a dozen linguistic adaptations, including the Chinese version [40]. Given this precedent, we opted to maintain the original scoring direction for item 9 in the present study. The observed negative correlation in the s-CRIq-C may reflect limitations in sample size or regional specificity; further validation with larger cohorts will be pursued. Thus, item 9 was retained with positive scoring in our study.

The findings indicated the strong reliability and validity of the s-CRIq-C. The s-CRIq-C demonstrated high concurrent validity with the full-length CRIq, supporting its potential as a valid alternative to the original Chinese version. Consequently, it is advisable to consider using the s-CRIq-C in future studies to enhance data collection efficiency and streamline study implementation processes. Reliability analyses revealed good internal consistency (Cronbach’s α = 0.777, McDonald’s ω = 0.780) and excellent two-week test-retest reliability (ICC = 0.968), outperforming the Chinese version of the CRIq (Cronbach’s α = 0.68, ICC = 0.87). These findings confirm that the s-CRIq-C possesses strong reliability and temporal stability, aligning with results from the original validation study [28].

### Limitations

This study has several limitations. First, the exclusive use of convenience sampling from the Ningxia region may limit the geographical representativeness of the sample and introduce potential selection bias, thereby limiting the external validity of the results. Second, using the full-length version (CRIq) as the primary criterion to validate the s-CRIq-C may involve potential circular reasoning. Future studies should employ external validation samples or objective cognitive assessments to further verify the validity of the s-CRIq-C. Finally, although the s-CRIq-C demonstrated strong validity, its self-report format is susceptible to biases such as social desirability bias (where participants may provide socially acceptable rather than truthful responses), limited self-awareness (leading to discrepancies between self-perception and actual states), and question interpretation variability (participants may understand the same items differently). Future studies could integrate objective measures with self-report data to further validate the findings.

## Conclusion

This study demonstrates that the s-CRIq-C exhibits good psychometric properties and offers benefits of ease of use, simplicity, and high efficiency within the Chinese adult population. Furthermore, it can serve as a reference for clinical evaluation and is recommended to support the early identification and intervention development for cognitive impairment-related disorders such as Alzheimer’s disease, mild cognitive impairment, and stroke, with the ultimate goal of mitigating cognitive decline and slowing disease progression. While the study acknowledges certain limitations discussed above, the s-CRIq-C shows promise as a valuable tool for future research and clinical applications.

## Data Availability

The datasets generated and analyzed during the current study are not publicly available to preserve anonymity of the respondents, but can be obtained from the corresponding author upon reasonable request. Additionally, the Chinese version of the s-CRIq can also be obtained from the corresponding author.

## Abbreviations

CR: Cognitive Reserve
CRI: Cognitive Reserve Index
CRIq: Cognitive Reserve Index Questionnaire
s-CRIq: Short version of the Cognitive Reserve Index Questionnaire
I-CVI: Item-level content validity index
S-CVI: Scale-level content validity index
S-CVI/UA: Scale-level Content Validity Index/Universal Agreement
S-CVI/Ave: Scale-level Content Validity Index/Average
TVI: Translation validity index
S-TVI: Scale-level translation Validity Index
ICC: Intraclass correlation coefficient
